# Efficacy and Safety Analysis of Ultrasonic Bone Curette in the Treatment of Thoracic Spinal Stenosis

**DOI:** 10.1111/os.12581

**Published:** 2019-12-10

**Authors:** Xiang‐dong Lu, Yi‐bo Zhao, Xiao‐feng Zhao, De‐tai Qi, Xu Yang, Xiao‐nan Wang, Run‐tian Zhou, Yuan‐zhang Jin, Bin Zhao

**Affiliations:** ^1^ Department of Orthopaedics the Second Hospital, Shanxi Medical University Taiyuan China

**Keywords:** Thoracic spinal stenosis, High‐speed drill, Posterior thoracic decompression, Ultrasonic bone curette

## Abstract

**Objective:**

To investigate the efficacy and safety of ultrasonic bone curette in treating thoracic spinal stenosis.

**Methods:**

A total of 30 patients of thoracic spinal stenosis who underwent posterior thoracic decompression in the hospital from December 2015 to 2017 were enrolled. Of these, 18 patients (group A) underwent posterior thoracic decompression using ultrasonic bone curette; and 12 patients underwent the treatment using a high‐speed drill (group B). The time of laminectomy, amount of intraoperative blood loss, presence or absence of cerebrospinal fluid leakage, and nerve root injury were recorded. All patients underwent X‐ray, computed tomography with three‐dimensional reconstruction, and magnetic resonance imaging before and after surgery. The Frankel classification and the Japanese Orthopaedic Association (JOA) scores were used to assess the neurological function and neurological recovery in patients. The measured data were statistically processed and analyzed using SPSS21.0 software, and the measurement data were expressed as mean ± SD.

**Results:**

In groups A and B, the average time for single‐segment laminectomy was 3.3 ± 1.2 min and 6.0 ± 1.8 min and the mean bleeding volume was 105.5 ± 43.3 mL and 177.4 ± 54.7 mL, respectively, with a statistically significant difference between the groups. The difference in JOA scores before and after surgery in groups A and B was statistically significant. No significant difference was found between the groups, in group A, the improvement rate of nerve function at the last follow‐up was 71% and in group B, the improvement rate at the last follow‐up was 70%. In group A, at last follow‐up, two patients had Frankel grade B injury, one had grade C injury, seven had grade D injury, and eight had grade E injury. In group B, at last follow‐up, one patient had Frankel grade B injury, one had grade C injury, five had grade D injury, and five had grade E injury. The Frankel classification of both groups A and B significantly improved. Four patients experienced cerebrospinal fluid leakage in group A and five in group B, with no significant difference between the groups. There was no nerve root injury in both groups, and no complications, such as pulmonary infection and urinary tract infection, occurred after operation.

**Conclusions:**

With the use of ultrasonic bone curette in posterior thoracic decompression, the decompression surgery could be completed relatively safely and quickly. It effectively reduced the amount of intraoperative blood loss.

## Introduction

Patients with thoracic spinal stenosis have an insidious onset, often accompanied by other spinal diseases, and are easily misdiagnosed with lumbar spinal stenosis and cervical spondylosis^1^. The course of the disease is often progressive, and conservative treatment is often ineffective, but it delays the condition^2^. Many patients have experienced severe neurological damage at the time of treatment. Therefore, once the patient has neurological symptoms, combined with the patient's clinical symptoms and signs, related image examinations, neuroelectrophysiological examination to identify the responsible segment, in order to save the patient's nerve function, and surgery should be performed as soon as possible.

At present, the thoracic spinal canal surgery includes anterior decompression, posterior decompression, circular decompression, and so on^3^, ^4^, of which posterior decompression is the most common procedure for the posterior approach. This type of procedure does not directly involve the calcification of the ligamentum flavum and the thoracic cord, it can achieve good clinical results by direct or indirect decompression, and the decompression effect is evident^5^, ^6^. However, the incidence of intraoperative complications is high and it is easy to damage the structure of the dura mater, nerve roots, spinal cord, and so on, which usually requires high surgical skills and tools^7^, ^8^.

Compared with the cervical spinal and lumbar spinal canals, the volume of the thoracic spinal canal is smaller. The thoracic spinal cord is the watershed area of the spinal cord circulation, and its blood supply is relatively small, which easily leads to spinal cord degeneration or ischemia–reperfusion injury^4^. If calcification and hypertrophy of the posterior longitudinal ligament or the ligamentum flavum are found, the original narrow vertebral canal hardly leaves space for the operation and for the buffer between the spinal canal wall and the spinal cord. In addition, studies have shown that dural adhesion, rapid progression, dural ossification, high signal in the spinal cord, multi‐segment lesions, irregular shape of the ossification of the ligamentum flavum will increase the risk of complications, therefore, the risk of thoracic spinal canal surgery is extremely high^8^. Therefore, how to remove the posterior lamina also derived from various procedures, such as the traditional “silkworm erosion” method using lamina biting forceps for laminectomy and decompression, stratified thinning of the posterior wall resection, removal of the posterior wall of the thoracic spinal canal using a high‐speed drill, and the technique of “cap uncovering”^9^, ^10^, ^11^. Traditional surgical bone chiseling removes the posterior wall of the spinal canal, which easily causes concussion of the spinal cord; also, the postoperative rate of paraplegia is high. The lamina is removed using lamina biting forceps in the “silkworm erosion” method. Although the lamina biting forceps are very thin, they still need to enter the dura mater and the lamina many times, which inevitably exerts downward pressure on the spinal cord and damages the spinal cord and nerve roots. At the same time, the workload of the operator greatly increases, the operation time is long, and the incidence of postoperative complications is high. However, a high‐speed drill greatly reduces the workload of the operator and shortens the operation time, but it relies on the friction with the vertebral lamina to consume bone mass. During the operation, the high‐speed drill bit rotates at a high speed, with large inertia, high heat production, and poor handle handling, easily scraping the surrounding soft tissue, injuring the spinal cord and increasing the risk of surgery^12^. The drill has a large volume, and the high‐speed rotary drill penetrates into the room of the lateral spinal canal wall and the spinal cord, easily causing compression of the spinal cord and scraping the surrounding soft tissue while cutting the lamina^13^, ^14^. Therefore, attempts are being made to find a more convenient and safe new surgical tool. Since the introduction of ultrasonic aspirator for the removal of dental plaque in 1947, ultrasonic instruments have gradually expanded to other fields. Ultrasonic bone curette has its unique characteristics. It is being increasingly used in orthopaedic surgery because its osteotomy is accurate and efficient and heat production is relatively low^15^.

Through retrospective comparative analysis of the clinical data of 30 patients with thoracic spinal stenosis treated by high speed drill or ultrasonic bone curette, the purpose of this study is: (i) to study the safety of ultrasonic bone curette in posterior thoracic laminectomy; (ii) to explore the surgical techniques and precautions of using ultrasonic bone curette; and (iii) to evaluate the clinical efficacy and prevention of complications of ultrasonic bone curette in posterior thoracic laminectomy.

## Materials and Methods

### *General Information*

A total of 30 patients were admitted to the hospital from December 2015 to 2017 who were diagnosed clearly by clinical symptoms and signs, image examination, and neuroelectrophysiological examination, and were treated by posterior thoracic decompression. Of these patients, 18 (group A: 13 men and 5 women, aged 45–73 years, with a mean age of 58.3 years) underwent posterior thoracic decompression using ultrasonic bone curette. The preoperative Japanese Orthopaedic Association (JOA) score was 4.2 ± 2.0 (1.0–7.0). Moreover, three patients had Frankel grade A injury, five had grade B injury, four had grade C injury, and six had grade D injury. The remaining12 patients (group B: five men and seven women, aged 45–67 years, with a mean age of 55 years) underwent the treatment using a high‐speed drill. The preoperative JOA score was 3.9 ± 2.1 (1.0–7.0). Moreover, two patients had Frankel grade A injury, five had grade B injury, one had grade C injury, and four had grade D injury.

### *Inclusion and Exclusion Criteria*

#### *Inclusion Criteria*

The inclusion criteria were as follows: (i) patients met the symptoms and signs of thoracic spinal stenosis, confirmed by imaging and neurophysiological examinations; (ii) patients treated with posterior thoracic decompression using ultrasonic bone curette or high‐speed drill; and (iii) patients who completed preoperative and postoperative imaging examinations and follow‐up; (iv) the main evaluation indicators included intraoperative blood loss, presence or absence of cerebrospinal fluid leakage, and nerve root injury, Frankel classification and the Japanese Orthopaedic Association (JOA) scores; and (v) a retrospective comparative study.

#### *Exclusion Criteria*

The exclusion criteria were as follows: (i) patients with heart, brain, kidney, and other important organ diseases; (ii) patients with thoracic spinal stenosis caused by acute trauma, spinal canal occupying lesions, and infection; and (iii) patients unable to cooperate with surgical treatment.

### *Clinical Manifestations and Imaging Features*

Patients in groups A and B had typical symptoms of thoracic spinal stenosis, such as numbness and weakness in both lower extremities. In group A, 10 patients had chest and back band feeling (55.6%), 14 had hypoesthesia (77.8%), 11 had positive ankle clonus and Babinski sign (61.1%), and 15 had double lower tendon hyperreflexia (83.3%). In group B, 4 patients had chest and back band feeling (33.3%), 8 patients had hypoesthesia (66.7%), 7 patients had ankle clonus (58.3%), 9 patients had a positive Babinski sign (75%), and 9 patients had double lower tendon hyperreflexia (75%).

All patients underwent thoracic vertebrae X‐ray, thoracic computed tomography (CT) with three‐dimensional reconstruction, and thoracic magnetic resonance imaging (MRI). Thoracic CT and MRI showed that both groups had hypertrophy of the ligamentum flavum with varying degrees of calcification. In group A, 12 patients had three or fewer segments involved, four had four segments involved, one had five segments involved, and one had 10 segments involved. In group B, eight patients had three or fewer segments involved, two had four segments involved, and two had five segments involved.

### *Surgical Methods*

**Figure 1:**
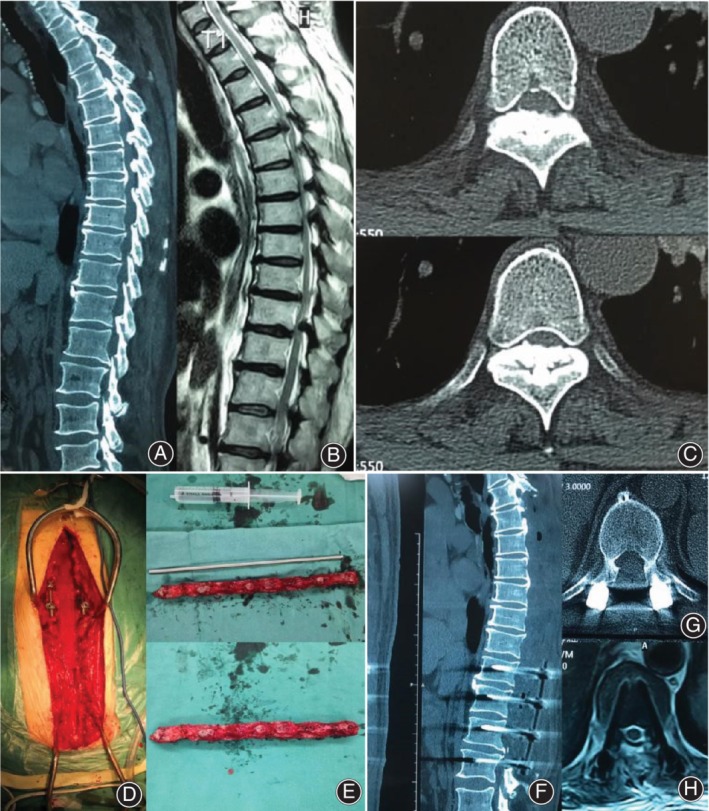
A 58‐year‐old male with numbness and weakness of both lower limbs for 3 years was diagnosed with thoracic spinal stenosis. (A and B) Preoperative MRI T2‐weighted images showed multisegment ligamentum flavum hypertrophy and corresponding spinal stenosis. Preoperative CT sagittal position showed multisegment ligamentum flavum calcification. (C) Preoperative thoracic CT axial position showed ligamentum flavum calcification and significantly reduced spinal canal volume. (D and E) Intraoperative thoracic spinal segment laminectomy resection. (F) Postoperative CT sagittal position chest four lumbar two laminectomy, parallel internal fixation to stabilize the thoracolumbar spine. (G and H) Postoperative thoracic CT MRI showed that the spinal cord was relieved, and the cerebrospinal fluid signal seen before and after the spinal cord was removed.

After general anesthesia, the patients were turned to a prone position, the chest and back skin were routinely disinfected, and aseptic surgical drape was laid. A median incision was made; the skin, subcutaneous tissue, and fascia were cut; the paraspinal muscles on both sides of the spinous process were removed; and the bilateral transverse process and lamina were exposed. Bone biting forceps were used to bite the spinous process of the responsible segment, and the whole layer of the bone was incised longitudinally along the middle line of the bilateral articular process using an ultrasound bone curette. Then, the head and tail laminae of the responsible segment were cut laterally; a bone knife was used to pry one side of the lamina, and the raised lamina was clamped with a towel clamp, and lifted like a cover. While picking up the lamina slowly, the nerve detachment was used to remove the ligamentum flavum and the dura mater. If the calcification and adhesion of the ligamentum flavum and dura mater were seriously damaged, some of the dura mater was removed. Then, whether the spinal cord was decompressed adequately was observed (Fig. 1).

The procedure in group B was the same as that in group A. First, the needle‐shaped rongeur was used to slot along the midline of the superior and inferior articular processes. Then, the high‐speed drill was used to thin the lamina along the slot, and lamina biting forceps were used to bite the thin lamina. Finally, the lamina was lifted using a bone knife and towel forceps. According to the number and location of decompression segments, internal fixation was used. An indwelling negative pressure drainage tube was left, and the incision was sutured layer by layer.

### *Observation and Follow‐up Indicators*

The time of laminectomy, intraoperative bleeding volume, and complications, such as cerebrospinal fluid (CSF) leakage, were recorded in both groups. All patients were outpatients followed up with an average follow‐up time of 14.1 ± 2.1 months. Neurological function was evaluated using Frankel grading and JOA spinal cord function score (full score 11). The improvement rate of neurological function was calculated as follows: improvement rate = (last follow‐up score ‐ preoperative score)/(11‐ preoperative score) × 100%.

#### *Frankel Grade for Spinal Cord Injury*

A: Complete loss of motor and sensory function; B: Incomplete ‐ preserved sensation only; C: Incomplete ‐ preserved motor (non‐functional); D: Incomplete ‐ preserved motor (functional); E: Complete return of all motor and sensory function, but may have abnormal reflexes.

#### *Japanese Orthopaedic Association (JOA) Score System*

The modified Japanese Orthopaedic Association (JOA) scoring system was used to evaluate the neurological status. The JOA score system includes four sections: lower‐limb motor dysfunction, lower‐limb sensory deficit, trunk sensory deficit, sphincter dysfunction. The maximum score of 11 indicates normal function. An improvement rate (IR) was calculated as: IR = (last follow‐up score ‐ pre‐operative score)/ (11‐pre‐operative score) × 100%. The IR was then used to define the surgical outcome: excellent (IR≥75%), good (75% > IR≥50%), fair (50% > IR≥25%) and poor (IR < 25%).

### *Statistical Analysis*

The measured data were statistically processed and analyzed using Statistical Program for Social Sciences 21.0 software (SPSS, Inc, Chicago, IL, USA), and the measurement data were expressed as mean ± SD. The time of intraoperative laminectomy, intraoperative bleeding volume, and neurological improvement were compared using two‐independent‐samples *t* test between groups A and B. A paired‐sample *t* test was used to compare the JOA scores before and after surgery, and the test level was *α* = 0.05. *P* value <0.05 was considered statistically significant.

## Results

There was no significant difference in gender, age, preoperative JOA scores and Frankel grade between the two groups (*P* > 0.05).

### *Surgery Information*

**Figure 2:**
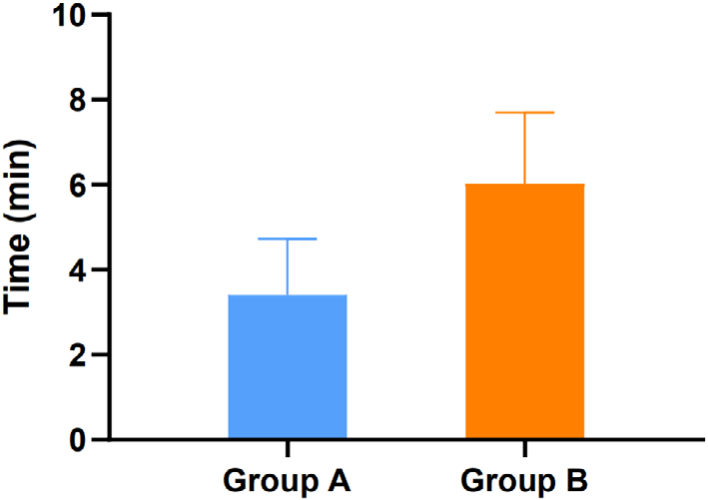
The single‐segment resection time: the time for single‐segment laminectomy was 3.3 ± 1.2 min and 6.0 ± 1.8 min for groups A and B.

**Figure 3:**
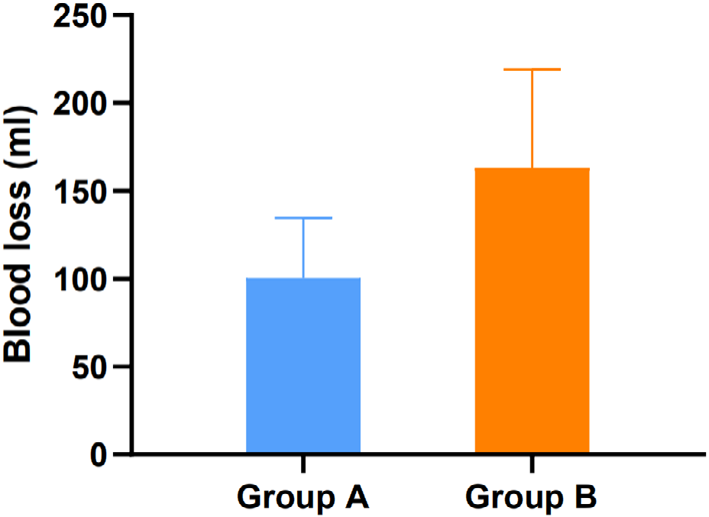
The single‐segment resection intraoperative blood loss, the bleeding volume was 105.5 ± 43.3 mL and 177.4 ± 54.7 mL for groups A and B.

In group A, 57 segments were resected; and in group B, 40 segments were resected. In groups A and B, the time for single‐segment laminectomy was 3.3 ± 1.2 min and 6.0 ± 1.8 min and the bleeding volume was 105.5 ± 43.3 mL and 177.4 ± 54.7 mL. The difference in single‐segment resection time and intraoperative blood loss between groups A and B was statistically significant (*P* < 0.05) (Table 1, Figs 2 and 3).

**Table 1:** Comparison between CSF leakage, single‐level laminectomy time, and bleeding volume between the two groups

Groups	Single‐level laminectomy time (mean±SD, min)	Single‐level laminectomy bleeding volume (mean±SD, mL)	CSF leakage (no. of patients)
Group A	3.3 ± 1.2	105.5 ± 43.3	4
Group B	6.0 ± 1.8	177.4 ± 54.7	5

### *Neurological Function Information*

#### *JOA Score and Improvement Rate*

**Figure 4:**
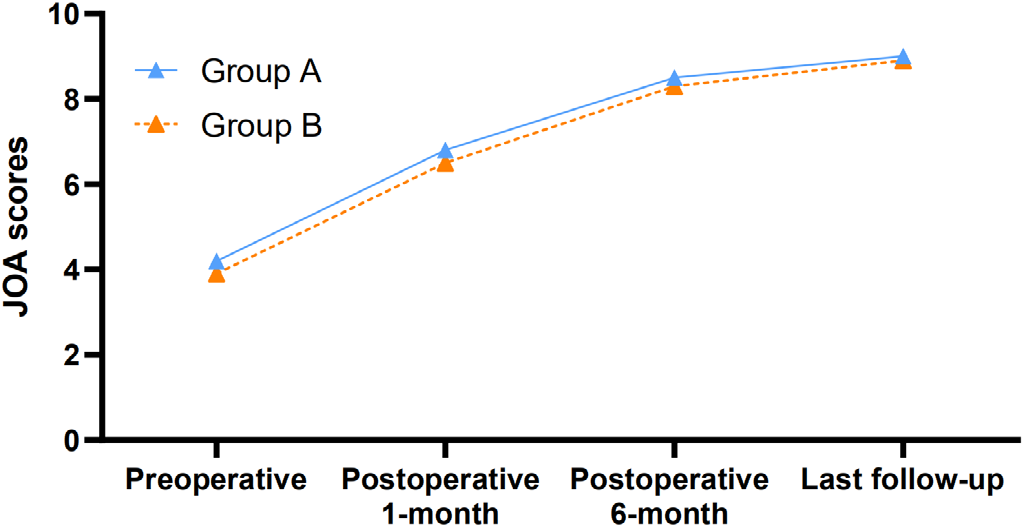
Preoperative and postoperative JOA scores in two groups. The postoperative JOA scores of patients in groups A and B were significantly higher than those before surgery, and significant differences in JOA scores before and after surgery were found in groups A and B (*P* < 0.05).

The postoperative JOA scores of patients in groups A and B were significantly higher than those before surgery, and significant differences in JOA scores before and after surgery were found in groups A and B (*P* < 0.05), with no significant difference between these two groups (*P* > 0.05) (Table 2, Fig. 4). In group A, the improvement rate of nerve function at the last follow‐up was 71%; in group B, the improvement rate of nerve function at the last follow‐up was 70%.

**Table 2:** Comparison of JOA scores between preoperative and postoperative 1‐month, 6‐month, and last follow‐up (maen±SD)

Groups	Preoperative	Postoperative 1‐month	Postoperative 6‐month	Last follow‐up
Group A	4.2 ± 2.0	6.8 ± 1.2	8.5 ± 1.1	9.0 ± 1.1
Group B	3.9 ± 2.1	6.5 ± 1.3	8.3 ± 1.4	8.9 ± 1.2

*Note*: In the two groups, the improvement in nerve function before and after surgery (*P* < 0.05), and the improvement in nerve function between the two groups were compared (*P* > 0.05).

#### *Frankel Grade*

**Figure 5:**
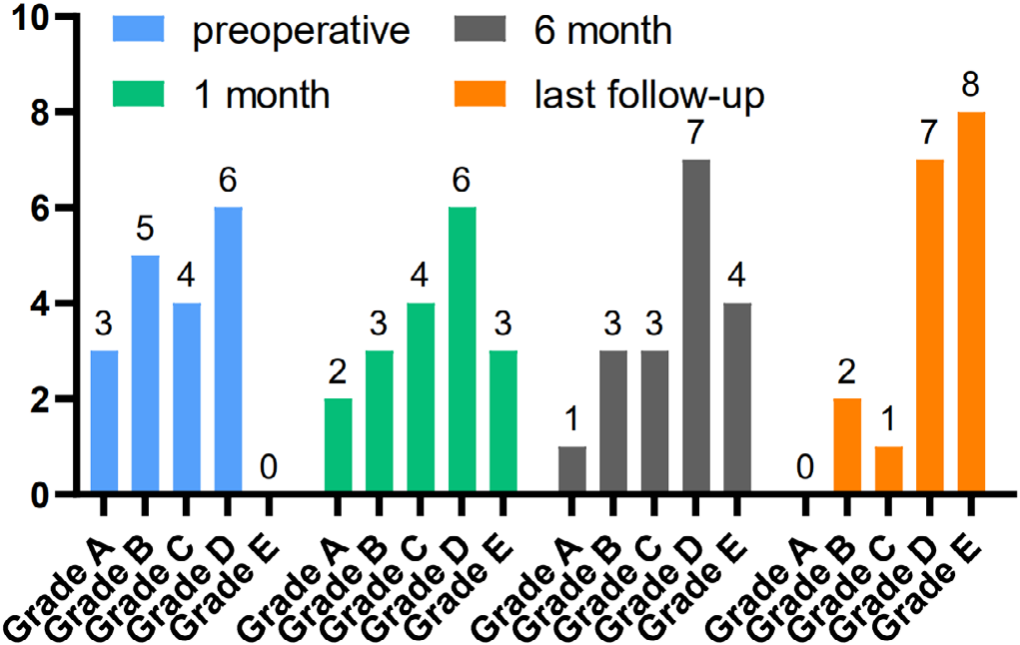
Frankel classification in group A: preoperation and postoperative follow‐up. Before surgery, three patients had Frankel grade A injury, five had grade B injury, four had grade C injury, and six had grade D injury; the last follow‐up, two patients had Frankel grade B injury, one had grade C injury, seven had grade D injury, and eight had grade E injury.

**Figure 6:**
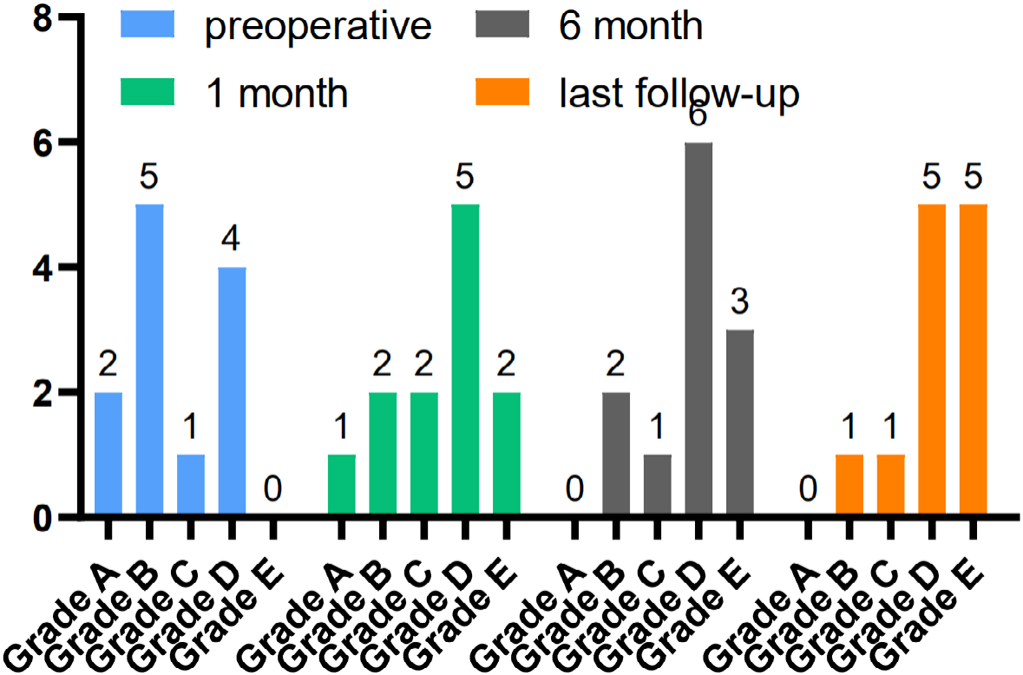
Frankel classification in group B: preoperation and postoperative follow‐up. Before surgery, two patients had Frankel grade A injury, five had grade B injury, one had grade C injury, and four had grade D injury; the last follow‐up, one patient had Frankel grade B injury, one had grade C injury, five had grade D injury, and five had grade E injury.

In group A, before surgery, three patients had Frankel grade A injury, five had grade B injury, four had grade C injury, and six had grade D injury; the last follow‐up, two patients had Frankel grade B injury, one had grade C injury, seven had grade D injury, and eight had grade E injury. In group B, before surgery, two patients had Frankel grade A injury, five had grade B injury, one had grade C injury, and four had grade D injury; the last follow‐up, one patient had Frankel grade B injury, one had grade C injury, five had grade D injury, and five had grade E injury. The Frankel classification of both groups A and B significantly improved (Figs 5 and 6).

### *Complications Information*

Four patients in group A and five patients in group B experienced cerebrospinal fluid (CSF) leakage, with no significant difference between the two groups (*P* > 0.05, Table 1). There was no nerve root injury in both groups, and no complications, such as pulmonary infection and urinary tract infection, occurred after operation.

## Discussion

### *Safety of Ultrasonic Bone Curette in Posterior Thoracic Laminectomy*

As a new surgical tool, ultrasonic bone curette has the following advantages: (i) the ability to distinguish bone from soft tissue. The vibration generated by the tip of the bone knife can identify the skeleton with specific hardness. Bone tissue can absorb more energy of the blade because of its greater rigidity, but the soft tissue structure (e.g., nerve and dura mater) absorbs less energy through reflective vibration and escapes the cutting of the ultrasonic bone knife^16^, ^17^; (ii) ultrasonic bone curette blade is thin and consumes less bone; when the blade enters the room of the lateral vertebral canal wall and spinal cord, the probability of dural injury is greatly reduced and the safety of operation is improved^18^. Ultrasound bone curette produces less heat compared with a high‐speed drill. Its own automatic cold‐water irrigation system can cool the knife blade, absorb heat, and, at the same time, make the operation field clearer^19^ and reduce the operational risk; (iii) during the process of cutting the lamina, the ultrasonic bone curette can play the role of stopping bleeding while cutting, reducing the bleeding of the cancellous bone and shortening the operation time; and (iv) the handle is easy to hold, has good manipulation, and reduces the labor intensity of the operator.

### *Surgical Skills and Precautions in Using the Ultrasonic Bone Curette*

When using the ultrasonic bone curette, it is necessary to control the cutting direction with one hand and antagonize it with another hand. Both hands should control the cutting depth together. When cutting the whole lamina, a sense of emptiness is experienced. At this time, the ultrasonic bone curette should be controlled to avoid the blade entering into the vertebral canal. A one‐time incision of the full lamina or ligamentum flavum should not be pursued; repeated careful operation is required. If necessary, lamina biting forceps should be used^20^ to prevent calcified dura mater, ligamentum flavum, and lamina from sticking to each other, resulting in dural tear and CSF leakage. This study found that complications of CSF leakage occurred in both groups A and B; therefore, a cautious operation is necessary. Although the heat generated by the ultrasonic bone cutter is lower than that produced by the high‐speed grinding drill, it still generates more heat when a large amount of bone is removed or the bone is hard, leading to prolonged operation of the cutter in the same part. To prevent the heat generated from stimulating the dura mater or spinal cord, the cutter should be operated intermittently^21^, ^22^.

### *Clinical Efficacy and Prevention of Complications of Ultrasonic Bone Curette in Posterior Thoracic Laminectomy*

The present study found that, compared with the high‐speed drill, the ultrasound bone curette not only achieved significant clinical efficacy but also shortened the laminectomy time, reduced intraoperative bleeding, and simplified the procedure, and reduced the risk of surgery.

In addition, in this study, four cases of cerebrospinal fluid leakage occurred in the ultrasonic bone curette group, and five cases of cerebrospinal fluid leakage occurred in the high‐speed drill group. No significant difference in the CSF leakage was found between the two groups; according to the recorded data, the probability of CSF leakage significantly reduced using the ultrasound bone curette (22.2% in group A and 41.7% in group B). Once cerebrospinal fluid leakage is found during the operation, it can be filled with back fat and gelatin sponge to keep the dura mater under certain pressure. After the operation, the bed tail is raised, the incision is drained smoothly, nutritional support treatment, especially protein supplementation and anti‐inflammatory treatment are done well. After the incision healed completely (12–14 days), the drainage tube was removed and the drainage port was changed for 3–5 days while healing.

### *Limitations*

The sample size in this study was relatively small. Therefore, future studies should compare the data of more patients. Also, the postoperative efficacy needs further long‐term follow‐up.

In conclusion, the ultrasonic bone curette was effective in posterior thoracic decompression, significantly reducing the operation time, intraoperative blood loss, and probability of damage to the spinal cord, nerve root, and dura mater. However, the risk of operation was still high, requiring the surgeon to have superb surgical skills and rich clinical experience, careful operation, and close observation of patients' condition and activity after the surgery.
